# Finding people who will tell you their thoughts on genomics—recruitment strategies for social sciences research

**DOI:** 10.1007/s12687-014-0184-2

**Published:** 2014-02-18

**Authors:** A. Middleton, E. Bragin, M. Parker, on behalf of the DDD Study

**Affiliations:** 1 Wellcome Trust Sanger Institute, Cambridge, CB10 1SA UK; 2 The Ethox Centre, Nuffield Department of Population Health, University of Oxford, Oxford, UK

**Keywords:** Survey, Attitudes, Social media, Facebook, Genomics, Ethics

## Abstract

This paper offers a description of how social media, traditional media and direct invitation were used as tools for the recruitment of 6,944 research participants for a social sciences study on genomics. The remit was to gather the views of various stakeholders towards sharing incidental findings from whole genome studies. This involved recruiting members of the public, genetic health professionals, genomic researchers and non-genetic health professionals. A novel survey was designed that contained ten integrated films; this was made available online and open for completion by anyone worldwide. The recruitment methods are described together with the convenience and snowballing sampling framework. The most successful strategy involved the utilisation of social media; Facebook, Blogging, Twitter, LinkedIn and Google Ads led to the ascertainment of over 75 % of the final sample. We conclude that the strategies used were successful in recruiting in eclectic mix of appropriate participants. Design of the survey and results from the study are presented separately.

## Introduction

### Setting

The Deciphering Developmental Disorders (DDD) project aims to discover new genetic diagnoses for children with developmental disorders in the UK (Firth et al. 2011). This involves the analysis, via exome sequencing, of each child’s 20,000 or so genes. The process of looking through thousands of genes in search for a diagnosis affords the opportunity to peruse genes known to be totally unrelated to the developmental disorder. Whether to look—or not—at such genes raises profound ethical dilemmas. These form the heart of the Genomethics research project (Middleton et al. 2013) which aimed to gather attitudes from all stakeholders about the deliberate choice to search for such ‘incidental findings’. Stakeholders included members of the public (who may be recipients of genomic sequencing technologies), genomic researchers (who may actually do the genomic sequencing) and health professionals, including genetic health professionals (who are familiar with working with individuals affected by and concerned about inherited conditions).

We created a novel online survey that contained ten integrated films (see www.genomethics.org). The films provided the background and contextual information needed in order to be able to answer the questions. The survey was designed so that it would be interesting and engaging to a whole spectrum of people, ranging from those who possibly knew nothing about genomics, e.g. members of the public, through to experts in the field, e.g. genomic researchers. The objectives of the overall study were to explore attitudes towards: (i) sharing of ‘pertinent findings’ from whole genome studies, (ii) sharing of ‘incidental findings’ from whole genome studies, (iii) receiving genetic information in different categories, (iv) genetic risk perception, (v) sharing of raw genomic data, (vi) genomic researchers having a duty to search for incidental findings, (vii) who might filter genomic data, (viii) possible consenting procedures for genomic studies, (ix) socio-demographic information and how this might affect the above variables. The survey design process—including the validation techniques applied—has been published separately (Middleton et al. 2014). Study results on the findings from just under 7,000 participants will also be published separately.

In this paper we outline and critically reflect upon the extensive and eclectic strategy for recruitment of participants into the study and suggest that social media is a particularly successful tool for participant ascertainment into genetics social sciences research.

### Overview of recruitment methods in use by others

Recent research exploring attitudes towards the sharing of incidental findings from genome studies have used various recruitment techniques. Those that have involved gathering the attitudes of researchers and health professionals have been done by directly inviting participation using professional email listserves or professional group membership (Ferriere and Van Ness 2012; Townsend et al. 2012; Downing et al. 2013; Fernandez et al. 2013; Klitzman et al. 2013). Members of the public participating in Focus Groups on their attitudes towards sharing incidental findings were recruited using advertisements in local newspapers, flyers and word of mouth (Haga et al. 2012; Townsend et al. 2012). Whilst not specifically on incidental findings Facebook has been used successfully in the recruitment of participants into other research about genetics (Reaves and Bianchi 2013), in particular direct to consumer genetic testing (McGuire et al. 2009; Leighton et al. 2012) and the experience of support gained from social networks for families with children with Trisomy 13 and 18 (Janvier et al. 2012). Twitter has been used successfully as a recruitment method in research that explored the experience of older mothers with regards to their pregnancy and birth and their attitudes towards non-invasive pre-natal diagnosis (O’Connor et al. 2013). Facebook adverts have been used as a recruitment tool to identify eligible low-income participants for a study on nutrition (Lohse 2013) and also young adults for a research project on substance use (Ramo and Prochaska 2012). Social media is increasingly being used in other areas of non-genomic social sciences research, and Facebook in particular has been identified as an important tool for recruitment into psychosocial research about genetics (Reaves and Bianchi 2013).

### Recruitment methods we chose to explore

Early on in the study design process we made the decision to collect our quantitative data via an online rather than postal survey (Middleton et al. 2014). This meant that irrespective of the recruitment strategy employed, it would only be accessed via the Internet.

1. Use of the Internet
  According to the Office of National Statistics in the UK, 86 % of the British population (43.6 million adults) have access to the Internet (Office for National Statistics 2013a), and 73 % (36 million) adults access the Internet every day (Office for National Statistics 2013b). Worldwide, 34 % of the population have access to the Internet, with usage least in Africa and highest in North America (Internet World Stats 2012). Social networking sites are used by 72 % of adults who are online (Brenner and Smith 2013). The age group of users that has seen the most significant growth has been amongst the over 65 s, with their presence tripling over the last 4 years from 13 % in 2009 to 43 % in 2013 (Brenner and Smith 2013). Thus, the Internet provides access to a worldwide convenience sample for any sort of research. By its very nature, enabling electronic connections to be made between users means it is also ripe for snowball sampling. It is for these reasons that we chose this as our medium for delivery of the survey.
2. Social networking
  Signposting potential research participants to the survey could be done via any number of strategies, and before recruitment started it was not possible to predict which method would be the most successful. As there are many social networking sites frequented by candidate research participants the decision was made to use an eclectic mix of the most popular sites: Facebook, Twitter and LinkedIn. A thorough review of what is available in terms of social media can be found in the following comprehensive text, ‘Blogging and other social media’ (Newson et al. 2008).
  1. Facebook was founded in 2004 by Mark Zuckerberg; it is a website that allows users to keep in touch with their friends, and people use it to share life events, photos and post messages. As of June 2013, it had 1.15 billion active users worldwide (Facebook 2013). Facebook connects people who have a personal or professional interest in genetics (e.g. American Society Human Genetics https://www.facebook.com/GeneticsSociety) but can also connect people who may have no specialist knowledge of genetics but just enjoy engaging in debate about interesting scientific issues (e.g. The Naked Scientists https://www.facebook.com/thenakedscientists). Searching for groups or individuals interested in genetics or genomics reveals millions of hits.
  2. Twitter was created in 2006. It is a website that enables users to send ‘tweets’ or text messages that contain 140 characters or less. As of September 2013, Twitter had 200 million users sending 400 million daily tweets (TECHi 2013). Daily conversations that cover issues relating to genetics are prolific; almost every permutation of discussion is possible, e.g. genomic researchers discussing the latest sequencing platforms search Twitter using #NGS, through to members of the public exploring a genetic diagnosis, see #geneticcondition.
  3. LinkedIn is a networking site for professionals. It allows colleagues to connect to each other and share information about work and professional interests; launched in 2003, it now has 200 million users worldwide (TECHi 2013). Most professional bodies and private companies linked to genetics now have LinkedIn groups, e.g. American Society Human Genetics, Illumina, National Society of Genetic Counselors.

Social media and traditional media are often directly linked. For example, television news outlets usually have an online presence as well as a Twitter feed. Each individual online news story can also typically be linked directly to personal social media feeds. Thus, it is possible to affect the momentum of social media by linking into traditional media sources such as TV and radio; in a cyclical motion, each feeds the other.

The following Methods section summarises the processes that were followed for recruitment, and the Results section provides details about the sample obtained.

## Material and methods

### Overview of methods

The overall study adopted a mixed methods approach, utilising both quantitative and qualitative techniques. For the quantitative arm, non-parametric data were gathered via 32 closed questions and explored using descriptive statistics. A web-link to the online survey was made available via the Wellcome Trust Sanger Institute in Cambridge, UK; this could also be accessed through a web-page that described the background to the study (www.genomethics.org).

### Participants

The study aim was to recruit participants who were genomic researchers, genetic health professionals (e.g. clinical geneticists, genetic counsellors, etc.), non-genetic health professionals (e.g. surgeons, GPs, nurses, etc.) and members of the public.

### Survey design

An extensive discussion on the survey design process can be found in a separate publication (Middleton et al. 2014). Here details are provided about the background work which was done to iteratively create a robust questionnaire; this involved a Focus Group, five pilot studies, readability tests, test-retest reliability measures and numerous stages of face validity testing. The resultant survey includes 32-closed questions gathering mainly categorical, quantitative data.

### Recruitment strategy

**Fig. 1:**
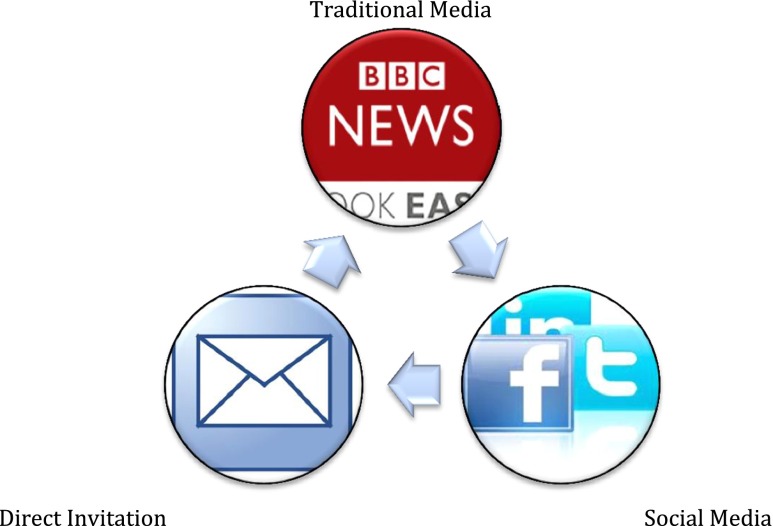
Three-phase interlinked recruitment strategy

A three-phase interlinked recruitment strategy was utilised (Fig. 1).

1. Traditional media
  Together with the media department at the Wellcome Trust Sanger Institute, a press release was written that advertised the study and invited participation. Following on from this, Channel 4 news and BBC local news created and delivered news stories on the research for the TV, and BBC Radio Cambridgeshire, BBC Radio 4 ‘Material World’ and the BBC World Service aired news stories for the radio. In each media article an interview with AM was conducted, and a link to the survey website was advertised. Each of these media also had an equivalent online news forum where a link to the survey was placed in an article summarising the project.
2. Direct invitation
  Members of various professional email listserves were invited directly by AM via email. Such groups included members of the Association of Genetic Nurses and Counsellors (AGNC), National Institute for Health Research (NIHR), Nuffield Council on Bioethics, Association of Medical Research Charities and staff from the Wellcome Trust Sanger Institute and The Wellcome Trust.
  Hard copies of flyers advertising the study and inviting participation were handed out directly to people attending the Royal Society Festival of Science, the Cheltenham Science Festival and at various genetics conferences the DDD team attended. They were also given directly to NHS professional recruiting into the molecular studies part of the DDD project. Such staff could also give these directly to patients attending clinic.
3. Social media
  AM worked with a Social Media Consultant to build the strategy for recruitment. The strategy involved the creation of an online infrastructure which comprised:
  Creating a brand and title: the word ‘Genomethics’ was invented—to represent the movement of the ‘genethics’ era (work on ethics and genetics) into the genomics era. One image was bought that symbolised the work; this was selected because it was considered user friendly enough to appeal to multiple audiences—a child playing with a DNA model. The image together with the title ‘Genomethics’ appeared on all the social media fora.
  A Facebook page was created called ‘Genomethics Survey’ (https://www.facebook.com/Genomethics). This offered a platform to disseminate the survey and create a list of followers who could do the same.
  A Twitter account was created: @Genomethics. This was used as a platform to enable participation in current debate about issues relating to genomics. It was also used as a tool to signpost potential participants to the survey.
  A ‘Genomethics’ website was created (www.genomethics.org) that contained information about the study and the survey. This was hosted at the Wellcome Trust Sanger Institute.
  A website for AM containing details of her CV and work on the genomethics study was created. This was to give credibility to the research, but in a ‘friendly’, ‘approachable’ way in-line with other social media mannerisms. This was constructed using www.wix.com (see www.annamiddleton.info).
  A LinkedIn profile was created for AM, containing the Genomethics brand image, plus CV details for AM. The purpose of this was to use professional networks to increase traffic to the survey.
  A Facebook ‘like’ button was added to the survey and so too was a Twitter share button so that participants could make their followers aware of the research.

All of the above media were used to create a robust infrastructure that could be used in multiple ways to advertise the survey and invite participation. This was specifically done using the following mechanisms.

### Blogging

The strategy focussed around the provision of blog posts that would opportunistically bring potential participants to the survey. AM designed a blog via www.wordpress.com (www.genomethicsblog.org) and periodically wrote short posts about various current issues being discussed within academic circles in genetics. The pieces were deliberately structured so that they would be appropriate for a mixed audience including those who knew nothing about genetics through to those currently working in the field. Within the article text—and also next to the article text—appeared a link and an image to the research survey. The intention was that, after reading the blog post, readers would serendipitously see and click on the survey.

Each Genomethics blog post was advertised on the linked Genomethics Twitter, Facebook and LinkedIn accounts. In each of these forums AM ‘chatted’ about the blog to encourage followers to link to it. AM also maintained a presence on Twitter, Facebook and LinkedIn, joining in with relevant discussions about genomics and signposting followers to related discussion—the ultimate aim of this was to increase the number of followers, thereby increasing the available audience who could ultimately access the blog and subsequently the survey,

AM also wrote blog posts for other providers, e.g. the Wellcome Trust, GenomesUnzipped, Cambridge Network, Swan (Syndromes Without a Name UK, a branch of Genetic Alliance UK), Cambridge Science Centre, Wellcome Trust Sanger Institute. For each of these articles a link was made to the Genomethics Twitter, Facebook and LinkedIn accounts. A link to the survey was also positioned on the landing page for the Decipher website, a site that hosts a consortium of ‘>200 academic clinical centres of genetic medicine and ≥1,600 clinical geneticists and diagnostic laboratory scientists’ (Bragin et al. 2013) and OMIM, which is a database used by clinical, medical and molecular geneticists worldwide (Baxevanis 2012).

### Google and Facebook adverts

**Fig. 2:**
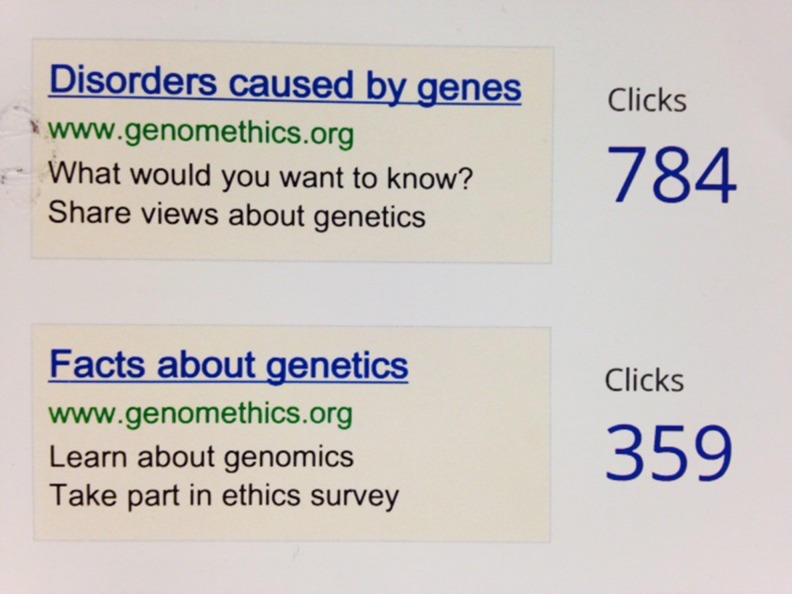
The two most successful adverts used on Google

A Google Ad account was opened by AM, and multiple advertisements for the survey were created. The adverts appeared each time specific terms were keyed into the Google search engine by any person using English. The advert appeared on the page, and viewers could choose to click on it; payment was taken per click. AM spent a long time researching the best terms to attach to each advert. Words such as ‘genome’, ‘ethics’ and ‘genetics’ are not popular and only used infrequently, whereas ‘disorders’, ‘mental illness’ and ‘genes’ were more popular search terms worldwide. Thus, these were chosen, and subsequently there were 549,566 appearances of several different adverts that contained various combinations of key words. Collectively, the adverts were clicked on 2,140 times (which cost £553 in total), and from this we received 215 completed surveys (i.e. approximately £2.50 per completed survey) (Fig. 2).

**Fig. 3:**
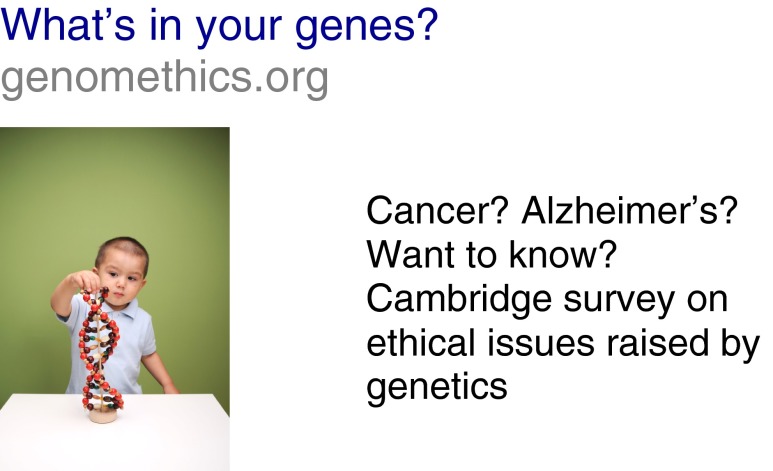
Facebook advert

A similar approach to above was used with Facebook. However, this time a research of appropriate search terms in Facebook revealed that ‘Cancer’, ‘Alzheimers’ and ‘Disorders’ were the most popular relevant terms, and so these were what were attached to the advert. The advert appeared 388,630 times on Facebook, and there were 259 clicks on it (at a cost of £76). It was not possible to determine how much traffic came to the survey directly from the advert or from the use of Facebook generally via other means, but in total we received 754 completed surveys via Facebook (Fig. 3).

### Advert in mumsnet and gransnet

Mumsnet is the UK’s biggest online network of parents. According to the site there are 50 million page views and 9 million visits per month. The Times newspaper reflects that it is ‘The country’s most popular meeting point for parents” (www.mumsnet.com). ‘Gransnet’ is a subsidiary website, particularly targeted towards grandparents. We wrote a short advert that Mumsnet and Gransnet put onto one of their pages for regular followers. It appeared as this:

*We’ve been asked by the Wellcome Trust Sanger Institute to ask Mumsnetters to fill in a survey they’re running on genetic testing.*

*Here’s what they say about the survey: “Your genes can tell you about your past, present and future medical health. Very soon, full genome testing (the ability to look at all 20,000+ genes) will be available in the health service. Like Angelina Jolie, you could have a genetic test and find out what you are at risk from. What would you want to know? Alzheimers? Cancer? Mental health issues? Or maybe you’d rather not know? Our research from Cambridge (* *www.genomethics.org* *) will have a direct impact on the way this testing is offered, find out about the possibilities and the ethical issues raised by this (no prior knowledge about genetics needed).”*

*The survey is open to everyone so please take part and pass on to any friends/family you think might be interested.*Please click here to take part.

Payment for the above advert cost £1,620, and we received 1,405 completed surveys; thus, each completed survey cost just over £1.

### Viral spread of survey

Due to the nature of the World Wide Web it is impossible to control how another user chooses to re-report and debate issues that the Genomethics project initiated. Other websites chose to write blogs based on our press release and wrote commentaries on the research on Facebook sites and via Twitter; participants also emailed their friends after completing the survey and ‘Liked’ it on Facebook and linked to it on Twitter. We had no influence on whether and how this was done, but the net effect was that a ‘viral’ or snowball process emerged whereby participants visited our website via routes completely unconnected to any of our active recruitment methods. For example, an online Polish newspaper ran an article on the study and provided a link to the survey (this was only discovered via an opportunistic google search). The net result of this was the direct recruitment of 90 new Polish research participants.

## Results

### Cleaning up of data

**Fig. 4:**
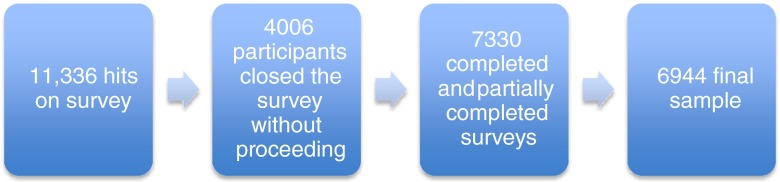
Compliance rate

The survey received 11,336 hits. Of these hits, 3,994 were immediately terminated, and the participant did not browse any further. No IP address was imprinted, and so there were no details that could define a profile of the non-responders. Of the participants who opened up the survey and had a look, 12 left the site without answering any questions. The remaining 7,330 completed or partially completed the questionnaire, 386 (5 %) dropped out of the survey after the first three questions (or appeared to give inconsistent answers throughout the survey, i.e. random button pressing) and the remaining 6,944 formed the final sample. Of these, 75 % of participants reached the last thank you message in the survey, and 72 % answered every question. See Fig. 4 for details. More specific details are provided in the publication written on the survey design process (Middleton et al. 2014).

There was no consistency in the questions that were missed out or partially answered. This indicated that once participants proceeded beyond the first three questions, the majority would continue the survey to the end, i.e. they were engaged enough in the survey to participate fully. Those who did pull out of the survey were the most likely to do this after the first three questions. The third question was: ‘Have you or your family ever been (or currently) a research participant in a genetic research project?’

### Profile of the participants who dropped out

There is very limited data on the participants who dropped out of the survey before the third question or gave inconsistent answers (i.e. apparent random button pressing), and no data at all on the 4,006 participants who closed the survey without proceeding and without answering any questions. However, we do have a simple profile of the background of the 386 participants who were removed from the final sample: 80 % were members of the public, 9 % were genetic health professionals, 7 % were non-genetic health professionals and 4 % were genomic researchers.

### Success of the recruitment

Table 1 shows how many participants were ascertained via each recruitment method.

**Table 1:** Success of each recruitment strategy

Strategy	Route	Completed surveys in final sample*	% of each recruitment method in final sample
Social media and the Internet	Google ads	215	4 %
	Facebook (inc Facebook ads)	754	14 %
	LinkedIn	14	0.5 %
	Twitter	183	3 %
	Solicited blogs (e.g. GenomesUnzipped, Cambridge Network, Unique, Swan, Sanger, Wellcome Trust, Cambridge Science Centre); Advert and link on OMIM and Decipher	92	2 %
	Advert in Mumsnet and Gransnet	1,405	26 %
	Word of mouth (inc unsolicited blogs based on press releases, online newspaper articles, participants who completed the survey then emailing their friends about it and teachers using the survey in teaching)	1,385	26 %
	**Total**	4,048	75 %
Traditional media	Press release picked up and articles on the research appearing on Channel 4 news, BBC news, Radio 4, Radio Cambridgeshire	455	9 %
	**Total**	455	10 %
Direct invitation	Email to AGNC, NIHR, Nuffield Council on Bioethics, Wellcome Trust, Sanger Institute	575	11 %
	DDD collaborators: handing out flyers, giving survey link to families and colleagues	233	4 %
	Handing out flyers at: Royal Society Festival of Science 2013, Cheltenham Science Festival 2011, Cambridge Science Festival 2012, handed out at local conferences	28	<1 %
	**Total**	836	15 %
	**Grand total for recorded recruitment strategy**	5339	

*Missing data on recruitment source = 1605

It is clear from Table 1 that social media was the most successful recruitment strategy out of the three used.

### Profile of the participants in the final sample

Tables 2, 3 and 4 show the breakdown of participant groups ascertained via each recruitment strategy. Missing numbers are not shown but can be deduced. This shows that social media (Table 2) was the most successful strategy for recruiting all four groups of participants (members of the public, genetic health professionals, non-genetic health professionals, genomic researchers).

**Table 2:** Profile of 455 participants recruited through traditional media

	Number	% of traditional media sample
Stakeholder group		
Genetic health professional	5	1 %
Genomic researcher	13	3 %
Other health professional	33	7 %
Public	404	89 %
Age		
20 or under	38	8 %
21–30	78	17 %
31–40	68	15 %
41–50	82	18 %
51–60	111	25 %
61 and over	78	17 %
Gender		
Female	238	52 %
Male	215	48 %
Prefer not to say	0	0
Marital status		
Married/civil partnership/living together	253	56 %
Divorced/separated/widowed/single	198	44 %
Ethnicity		
White	418	93 %
Afro-European, African American, Black	9	2 %
Hispanic	1	<1 %
South Asian Indian, Pakistani	8	2 %
East Asian Chinese, Japanese	1	<1 %
Arabic, Central Asian	2	<1 %
Other ethnicity	12	3 %
Education		
Completed primary school/preparatory school/elementary school	11	2 %
Currently studying at secondary school/high school	12	3 %
Completed secondary school/high school	100	22 %
Currently studying at university/college/tertiary education institution	44	10 %
Completed degree(s) at university/college/other tertiary education institution	264	58 %
Other education	22	5 %
Continent		
North America	10	2 %
Europe	443	97 %
Rest of world	2	<1 %

**Table 3:** Profile of 836 participants recruited through direct invitation

	Number	% of direct invitation sample
Stakeholder group		
Genetic health professional	166	20 %
Genomic researcher	193	23 %
Other health professional	75	9 %
Public	402	48 %
Age		
20 or under	25	3 %
21–30	205	25 %
31–40	253	30 %
41–50	175	21 %
51–60	102	12 %
61 and over	71	9 %
Gender		
Female	548	66 %
Male	278	33 %
Prefer not to say	7	1 %
Marital status		
Married/civil partnership/living together	564	68 %
Divorced/separated/widowed/single	272	32 %
Ethnicity		
White	744	90 %
Afro-European, African American, Black	8	1 %
Hispanic	11	1 %
South Asian Indian, Pakistani	25	3 %
East Asian Chinese, Japanese	13	2 %
Arabic, Central Asian	8	1 %
Other ethnicity	22	2 %
Education		
Completed primary school/preparatory school/elementary school	13	2 %
Currently studying at secondary school/high school	8	1 %
Completed secondary school/high school	70	8 %
Currently studying at university/college/tertiary education institution	55	7 %
Completed degree(s) at university/college/other tertiary education institution	644	77 %
Other education	43	5 %
Continent		
North America	56	7 %
Europe	763	92 %
Rest of world	14	1 %

**Table 4:** Profile of 4,048 participants recruited through social media

	Number	% of social media sample
Stakeholder group		
Genetic health professional	232	6 %
Genomic researcher	276	7 %
Other health professional	492	12 %
Public	3,048	75 %
Age		
20 or under	160	4 %
21–30	848	21 %
31–40	1,292	32 %
41–50	837	21 %
51–60	449	11 %
61 and over	428	11 %
Gender		
Female	3,126	78 %
Male	866	21 %
Prefer not to say	22	1 %
Marital status		
Married/civil partnership/living together	2,892	72 %
Divorced/separated/widowed/single	1,156	28 %
Ethnicity		
White	3,694	92 %
Afro-European, African American, Black	33	1 %
Hispanic	45	1 %
South Asian Indian, Pakistani	69	2 %
East Asian Chinese, Japanese	42	1 %
Arabic, Central Asian	18	0.4 %
Other ethnicity	127	3 %
Education		
Completed primary school/preparatory school/elementary school	52	1 %
Currently studying at secondary school/high school	49	1 %
Completed secondary school/high school	528	13 %
Currently studying at university/college/tertiary education institution	371	9 %
Completed degree(s) at university/college/other tertiary education institution	2,789	70 %
Other education	225	6 %
Continent		
North America	757	19 %
Europe	3,131	77 %
Rest of world	160	4 %

The above-mentioned tables also show that irrespective of recruitment source the majority of participants were aged 31–50, female, married, white, highly educated and from Europe.

## Discussion

Information about every aspect of personal and professional life is now available on the Internet; this is of course true for information about genetics and genomics. Collectively, genetic health professionals and genomic researchers use thousands of sites as reference points for information as well as spaces for discussion about issues pertinent to their industry, just to name a few: Decipher (www.decipher.sanger.ac.uk), GeneReviews (www.ncbi.nlm.nih.gov/books/NBK1116/) and GenomesUnzipped (www.genomesunzipped.org). People affected by genetic disease are able to easily access websites related to a condition or group of conditions, e.g. Unique (www.rarechromo.co.uk/html/home.asp) and GeneticAlliance (www.geneticalliance.org.uk). On Google the search term ‘genomics’ revealed 11 million hits; thus, the Internet offers an abundant data source and by virtue of this provides a rich viewing audience, ripe for collection for research assessing attitudes towards the use of genomics.

As interest in the survey could spread virally, i.e. people who enjoyed participating told their online friends about it, the sampling frame was thus both convenience and snowball. Due to this it was not possible to draw conclusions on whether the final sample was representative of the Internet population as a whole or indeed representative of any specific population.

The final ascertained sample consisted of participants who were predominantly female, white, highly educated and aged 31–50. Below is an exploration of whether this is a typical profile of people who take part in surveys as well as those who use social media, access traditional media such as news programmes and are part of the select professional groups targeted.

### Demographics of social networkers

It is very difficult to obtain accurate information on the generic profile of Facebook, Twitter and LinkedIn users as the rate of growth for these three media is phenomenal and each site rarely reports user demographic data. It is also surprisingly difficult to mine the Internet generally for up-to-date statistics about social media that are evidence based, collected via robust research methods; thus, the following information is provided only as a guide.

1. Age
  The most popular age range for social media users generally is 35–44 years (Macmillan 2011); 65 % of US Facebook users and 37 % of UK Facebook users are 35 or older (Pingdom 2012). According to Sakki (2013) Facebook users are more likely to be over 25 (Sakki 2013). The average Facebook user is thought to range from 18–29 years (Duggan and Brenner 2013), 25–34 years (Fanalyzer 2013), 38 years (Macmillan 2011) through to 40.5 years old (Pingdom 2012). For Twitter, 55 % of US users are 35 or older (Pingdom 2012), and most Twitter users in the UK are over 35; the age range is between 18 and 29 years (Sakki 2013), and average age is 37.3 years old (Pingdom 2012) and 39 years old (Macmillan 2011). For LinkedIn, 79 % of US users are 35 or older, and the majority of UK users are over 35 (Sakki 2013) with the average user being 44.2 years old (Macmillan 2011; Pingdom 2012).
  As Table 4 shows the 4,048 participants we recruited via social media were more likely to be in the 31–50 age range. Thus, our sample is typical of the ‘average’ user of social media as reported by other sources.
2. Gender
  Women are more likely to access social network sites compared to men (Emerson 2011; eMarketer 2013), and according to the UK’s Office of Communications (Ofcom) those women who do access social media sites do so more frequently than men (Ofcom 2013). Women also have 55 % more wall posts on Facebook than men (Boglioli 2011), and women spend, on average, 9 % more in terms of time on social networking sites generally than men (Widrich 2013).
  In the US 60 % of Facebook users are women (Pingdom 2012). In the UK 51 % of Facebook users are women (Fanalyzer 2013). In the US 60 % of Twitter users are women (Pingdom 2012), and for LinkedIn, 53 % are women (Pingdom 2012). Slightly different figures are given by Sakki in the UK, who report that Facebook is used in equal numbers by men and women, Twitter is used slightly more by women (51 % compared to 49 %) and LinkedIn is used more by men (58 % compared to 42 %) (Sakki 2013).
  Table 4 shows that our sample recruited through social media was predominantly female. This also fits with the generic profile data on social media use by gender as reported by other sources.
3. Household income, education, ethnicity and marital status
  The Pew Internet and American Life Project catalogues trends in social media use (www.pewinternet.org); this research relates to the American market and was taken from their latest survey in 2012. The average Facebook user is educated (73 % had some college attainment, and 68 % had completed college), with a household income above $75 k and living in urban areas (there was no data on ethnicity for Facebook; however, social media users generally were slightly more likely to be Hispanic or Black than White). Whereas the average Twitter user is African-American with some college education, with a household income above $75 k living in urban areas (Duggan and Brenner 2013). In the UK 69 % of Facebook users are in a relationship (Fanalyzer 2013). The majority of our sample recruited through social media were also in a relationship. Our sample was also overwhelmingly white (92 %), and there was little representation from other ethnic or racial groups. The vast majority of participants in the final sample were from Europe, and whilst this continent still consists of an eclectic mix of different ethnic and racial groups, the majority of people from Europe would still class themselves as white. We did not gather data on household income, but the profile of our users was of a very high level of academic achievement (70 % had a degree or higher level of education). Even if the health professionals and genomic researchers were removed from this calculation the research participants who are members of the public still selectively have a higher educational level than one might expect of a representative public.
  Whilst generically it appears that social media users may be more likely to have higher education levels than not, our sample was particularly biased towards the well educated. This may be due to a combination of factors—the subject matter may hold particular interest to those who have studied biology before or to those who are interested in ethical issues raised by technologies. In addition to this research shows that participating in surveys is more likely to draw educated people than other groups (Curtin et al. 2000; Singer et al. 2000; Goyder et al. 2002), and also online surveys particularly about genetics have a tendency to draw an educated crowd (Reaves and Bianchi 2013).
  Whilst it is not possible to provide robust calculations as to whether the convenience sample gathered via social media is in any way representative of generic users of social media, it does appear that the sample is typical of users of this medium.

### Demographics of people who use traditional media

Despite an extensive literature and online search it was not possible to unpick a typical demograph of a person who is likely to respond to a British television news article, let alone one specifically on genetics. However, research from the US shows that viewers of evening news programmes have consistently been on the decline, and this is particularly true of younger age groups (Guskin et al. 2011). The average evening news consumer in the US is over 50, female, with a higher than average level of education and a household income of greater than $75 k and education (Pew Research Center 2012). The sample ascertained via the Traditional Media recruitment method was more likely to be over the age of 41, female and highly educated; this does broadly fit with the profile identified from the American research (which is subtly different from the social media group).

### Demographics of people accessed via direct invitation

There is no published publically available data on the demographics of staff approached directly via email listserves to participate in our survey, i.e. from the AGNC, NIHR, Nuffield Council on Bioethics, Wellcome Trust Sanger Institute, Wellcome Trust and Association of Medical Research Charities. However, as a member of the AGNC the first author is aware anecdotally that the majority of genetic counsellors in the UK are female, white, highly educated and aged 31–50. It was not possible to document the demographics of patients who picked up a flyer as part of their attendance at a Science Festival or NHS appointment. What is known, however, is that the demographic data provided in Table 3 largely fits the same demographic data in Tables 2 and 4. It is therefore distinctly possible that the typical demograph of people we have recruited more broadly fits with the type of person who is just generically interested in participating in research about genetics. This leads us to an exploration of the literature already published on attitudes towards various issues surrounding genetics and whether there is a typical profile of participants who engage with this research.

### Demographics of people who take part in research about genetics

Research gathering attitudes towards the use of genetic technology have been conducted for over 20 years. Numerous types of participant groups have been sampled and studied; it is difficult to know whether there is a particular type of person who is more likely to be drawn to participate in research on genetics, but it is possible to explore the research that has been done and the socio-demographic data attached to the participants involved. The following studies are very typical examples from an enormous body of literature.

Kerath et al (2013) explored the beliefs and attitudes of members of the public towards participating in genetic research. The survey was distributed to a convenience sample of people attending a network of 15 different hospitals around New York. The sample supposedly represented the ‘diverse, geographic, socioeconomic and ethic catchment areas of the Health System’ (Kerath et al. 2013). Within their final sample (*n* = 1,041), the majority who chose to complete surveys were over 40, female, white, had a degree or graduate degree, were married and had children.

Cherkas et al. (2010) gathered British attitudes towards personal genome testing from 4,050 members of the public. Their survey was distributed to a convenience sample of twins participating in the TwinsUK Adult Twin Registry, who had been ascertained from the general population. The mean age of participants in the study about genetics was 56, 89 % were female, 79 % had children and the majority were of higher socio-economic status (Cherkas et al. 2010).

Morren et al. (2007) explored attitudes towards genetic testing amongst patients with chronic disease in The Netherlands. The survey was mailed to a nationwide representative sample of patients with chronic disease and returned by 1,496 participants. Within the final sample, the majority of participants were over age 45, 58 % of them were female, 75 % married/cohabiting and 54 % had an ‘intermediate’ or ‘high’ level of education (Wilde et al. 2010).

Whilst there are clearly numerous research projects on attitudes towards various issues in genetics that have been particularly focussed on gathering the views of men (Quinn et al. 2010), certain ethnic groups (Murphy and Thompson 2009, Ahmed, Ahmed et al. 2012) and specific ages of people (Donnelly et al. 2013) these are by far in the minority of the whole body of published work available.

When exploring the literature on the profile of nonresponders to surveys, an interesting Faculty paper was uncovered from William G Smith (2008) at the San Jose State University. Smith summarises the literature on the typical profiles of people who take part in survey research (Smith 2008). He showed that generally people who are educated and affluent are more likely to take part than less educated and less affluent people (Curtin et al. 2000; Singer et al. 2000; Goyder et al. 2002); women are more likely to participate than men (Curtin et al. 2000; Singer et al. 2000; Moore and Tarnai 2002) and white people are more likely to participate than other ethnic or racial groups (Curtin et al. 2000; Groves et al. 2000).

Therefore, the convenience and snowball sample that we have obtained via the three recruitment strategies broadly fit the samples that have been recruited for other research on genetics. The sample also fits with the profile of respondents who generically respond to recruitment invitations to participate in social sciences research. Separate publications will follow that will explore how socio-demographic data are linked to attitudes towards sharing incidental findings from genomics.

Future social science research on genomics could very usefully employ selective sampling frames that specifically target non-white audiences, men, as well as people who have lower educational achievements and affluence. It is only with the contribution of these other groups that useful conclusions can be more broadly drawn on attitudes towards the use of genomic technologies.
